# Characteristic of cerebral hemodynamics and functional connectivity in patients with neuropathic pain after spinal cord injury: an exploratory study

**DOI:** 10.3389/fnins.2025.1699161

**Published:** 2025-12-10

**Authors:** Yang Yu, Yuqin Ma, Meiling Cheng, Zebin Huang, Honghui Lei, Sitong Su, Fubiao Huang, Fangyong Wang

**Affiliations:** 1 Department of Spine Surgery, Beijing Bo'ai Hospital, China Rehabilitation Research Center, Beijing, China; 2 School of Rehabilitation, Capital Medical University, Beijing, China; 3 Department of Rehabilitation Medicine, Nanjing First Hospital, Nanjing, Jiangsu, China; 4 Rehabilitation Medicine Center, The Second Affiliated Hospital and Yuying Children's Hospital, Wenzhou Medical University, Wenzhou, China; 5 School of Biological Science and Medical Engineering, Beihang University, Beijing, China; 6 Department of Occupational Therapy, Beijing Bo'ai Hospital, China Rehabilitation Research Center, Beijing, China; 7 University of Health and Rehabilitation Sciences, Qingdao, Shandong, China; 8 School of Rehabilitation Medicine, Shandong University of Traditional Chinese Medicine, Jinan, China

**Keywords:** spinal cord injury (SCI), neuropathic pain (NP), functional near-infrared spectroscopy (fNIRS), oxyhemoglobin (HbO), functional connectivity (FC)

## Abstract

**Background:**

This study investigated the brain functional characteristics of patients with neuropathic pain (NP) following spinal cord injury (SCI) using functional near-infrared spectroscopy (fNIRS).

**Methods:**

A total of 35 subjects were enrolled, including 10 able-bodied controls, 12 patients with SCI and NP (SCI-NP), and 13 patients with SCI (without NP). fNIRS was used to detected blood oxygen signals during motor tasks and resting-state (RS) functional connectivity (FC) in the subjects. We also performed Pearson correlation analyses of pain scores (NPS) and the Pittsburgh Sleep Quality Index (PSQI) in patients with SCI-NP. Statistical analyses were performed using Shapiro-Wilk test for normality; paired *t-*test for intra-group differences; ANOVA (LSD *post-hoc*, Bonferroni-corrected) for multi-group comparisons; Cohen's d/partial η^2^ for effect size; independent samples *t*-test (FDR-corrected) for inter-group FC differences.

**Results:**

During the task state, patients with SCI-NP activated bilateral primary somatosensory cortex (S1, L/R *P* = 0.044/0.032), bilateral supplementary motor area (SMA, L/R *P* = 0.041/0.037), right primary motor cortex and left parietal lobe (LPL, *P* = 0.047). Hemodynamic changes in the bilateral S1, secondary somatosensory cortex, SMA, and right prefrontal cortex of patients with SCI-NP were significantly stronger than those in the other two groups. During RS, the whole-brain and most inter/intra-regional FC strength trended able-bodied controls > patients with SCI > patients with SCI-NP. NPS strongly correlated with LPL (r = 0.977, *P* = 0.004); PSQI correlated with motor cortex HbO changes (r = 0.889−0.975, *P* = 0.005−0.043).

**Conclusion:**

Patients with SCI-NP exhibit significant abnormal cerebral cortical excitation and reduced FC. HbO is a potential biomarker for evaluating NP. fNIRS supports objective assessment of SCI-NP and rehabilitation strategy formulation [ChiCTR2500097098].

## 1 Introduction

Neuropathic pain (NP) as a common high-incidence complication following spinal cord injury (SCI), has an incidence rate as high as 61% to 85% (Rosner et al., 2023). NP is a refractory chronic pain caused by nervous system injury or dysfunction, with clinical manifestations including persistent pain induced by mechanical or thermal stimulation, as well as paroxysmal spontaneous pain; it is also often accompanied by hyperalgesia (an enhanced pain response to noxious stimuli) and allodynia (pain induced by non-noxious stimuli) (Shiao and Lee-Kubli, 2018). These pain characteristics not only delay the rehabilitation process of patients but also significantly reduce their quality of life, while increasing the economic burden on families and society (Widerström-Noga, 2023).

The pathogenesis of NP has not been fully elucidated, and it is currently widely recognized that its occurrence is associated with dysfunction of inhibitory interneurons (Kang et al., 2020). Following SCI, GABAergic, and glycinergic inhibitory interneurons in the dorsal horn of the spinal cord undergo substantial loss or functional impairment due to ischemia, inflammation, or direct injury, resulting in weakened inhibitory effects on nociceptive signals. After the impairment of normal pain inhibitory pathways, peripheral nociceptive signals are excessively transmitted to higher centers such as the thalamus and cortex via ascending pathways including the spinothalamic tract, thereby triggering central sensitization—a phenomenon where the sensitivity of neurons to pain signals is persistently enhanced, such that mild stimuli can induce intense pain perception (Lütolf et al., 2022). In addition to excessive neuronal excitation after injury, studies have indicated that the development of chronic pain is also associated with sensorimotor cortex reorganization following injury-induced loss of motor and sensory functions (Li et al., 2023). Existing studies have confirmed that multiple structural and functional changes exist in the brains of patients with chronic pain, including abnormal brain networks, atrophy of multiple brain regions, and dysfunction of the endogenous pain modulation system (Ng et al., 2018). Multiple studies have evaluated the persistent characteristics of pathological chronic pain states by comparing brain structural and functional features between patients with chronic pain and healthy subjects. The results have revealed that chronic pain is closely associated with extensive structural and functional changes in the brain, involving multiple brain regions such as the prefrontal cortex (PFC) (Ong et al., 2019), insula (Cole et al., 2018), anterior cingulate cortex (Chen B. et al., 2025), and limbic structures (Singh et al., 2022). Currently, research on changes in brain function after SCI remains scarce; therefore, no clear conclusion has been drawn regarding whether similar structural and functional brain changes exist in patients with NP following SCI (SCI-NP).

Currently, clinical assessment of NP primarily relies on subjective self-reporting and clinical experience. Pain intensity is mostly evaluated using commonly used verbal rating scales or numerical rating scales (Attal et al., 2018), or through testing the patient's perception type, significance, and threshold of pain (Rosner et al., 2021). The assessment methods using mechanical, cold, and thermal stimuli, which are commonly applied in rodent models, are also applicable to patients with SCI (Kramer et al., 2017). However, these methods largely depend on spinal cord-mediated simple withdrawal reflexes. Moreover, patients with SCI-NP tends to occur below the level of injury, and there are varying degrees of limb dysfunction, resulting in insufficient sensitivity of withdrawal movements, thus leading to low standardization of these methods. Neuroelectrophysiological examinations can detect somatosensory evoked potentials (SEP) and spinal cord evoked potentials (SCEP). Nevertheless, SCI damages the integrity of sensory conduction pathways, making it impossible to accurately assess the nature and scope of pain (Liang et al., 2020). Electroencephalography (EEG) can also be used for brain connectivity analysis in patients with SCI-NP (Hasan et al., 2024; Kumari et al., 2022); however, this technique has limitations: on the one hand, it may induce scalp irritation or headache, and on the other hand, it is susceptible to interference from other electromagnetic signals, which in turn affects data accuracy. Neuroimaging techniques have potential advantages in the quantitative assessment of chronic pain. For example, positron emission tomography (PET) can identify brain regions involved in pain processing by studying the brain's response to evoked pain (Yoon et al., 2014); however, it is rarely used for pain detection due to its high cost. Traditional functional magnetic resonance imaging (fMRI) can measure patients with SCI-NP' brain network by assessing the brain's resting state (Mandloi et al., 2024; Shoraka et al., 2024). However, fMRI has numerous limitations in its application to clinical rehabilitation settings and large-sample studies (Menant et al., 2020): it has relatively low temporal resolution (only 1–2 seconds), which prevents it from capturing subtle dynamic changes in cerebral hemodynamics during tasks; even minor head movements can severely interfere with fMRI data and cause data distortion, which makes it difficult to apply fMRI to detection under motor state; the scanning process is relatively time-consuming, typically requiring 30–60 min; and the technique requires a specialized high-magnetic-field scanning environment, which is demanding. By contrast, functional near-infrared spectroscopy (fNIRS) can effectively address the aforementioned limitations.

Functional near-infrared spectroscopy (fNIRS) is a non-invasive brain function detection technique that can real-time detect hemodynamic fluctuations of oxyhemoglobin (HbO) and deoxyhemoglobin (HbR) in brain tissue, and thereby infer the underlying neural activity of the brain. This technique is characterized by significant advantages such as portability, non-invasiveness, almost no ionizing radiation, simple operation, high temporal resolution, and the ability to effectively handle motion artifacts (Sevick-Muraca, 2012). Currently, multiple fNIRS studies have confirmed its feasibility in *in vivo* research on pain perception (Luo et al., 2024).

fNIRS can identify and map cortical activation regions during painful stimulation. For example, Üçeyler et al. (2015) used fNIRS to investigate cortical hemodynamic activity in response to painful stimulation in patients with fibromyalgia, and found that activation of the bilateral dorsolateral prefrontal cortex (DLPFC) was significantly enhanced; the activation pattern of the sensorimotor cortex changes during movement in patients with knee osteoarthritis (Yang et al., 2024); HbO levels in the primary motor cortex (PMC) are increased in amputee patients with phantom limb pain (Simis et al., 2024). However, the aforementioned studies have not addressed the mechanisms of interaction between these brain regions. Functional connectivity (FC) is defined as the correlation of spontaneous fluctuations in neural activation patterns across different brain regions, serving as the basis for information transmission within brain networks (Hu et al., 2018). Previous traditional fNIRS studies have shown that chronic pain can lead to enhanced or weakened functional connectivity in specific brain regions (Yuan et al., 2016); resting-state (RS) FC has been applied in newly diagnosed patients with subacute back pain to distinguish individuals who eventually develop chronic back pain from those who do not (Baliki et al., 2012). All the aforementioned studies demonstrate the application of fNIRS in detecting brain function in chronic pain; however, relevant research on SCI-NP remains scarce to date.

Therefore, the present study aims to detect task-state blood oxygen signal changes and RS FC characteristics in patients with and without NP following SCI. On the basis of exploring the pathogenesis of NP and brain function changes in these patients, it further investigates the feasibility of HbO as a biomarker for patients with SCI-NP, as well as the application value of fNIRS as an objective examination method for NP. This is an exploratory study that intends to provide a theoretical basis and practical reference for pain assessment and the formulation of rehabilitation treatment strategies in patients with SCI-NP.

## 2 Methods

### 2.1 Participants

A total of 35 participants were recruited in this study, including 10 able-bodied controls and 25 patients with SCI. Specifically, 25 patients with SCI who were admitted for rehabilitation treatment in the Department of Spinal Surgery, Beijing Bo'ai Hospital, China Rehabilitation Research Center (CRRC) from March 2025 to June 2025 and met the inclusion and exclusion criteria were enrolled. The 10 able-bodied controls were doctors and postgraduate students from the same department. The study protocol was approved by the Medical Ethics Committee of CRRC (No. 2024-106-01) and conducted in accordance with the ethical principles of the Declaration of Helsinki (revised 2008).

Inclusion criteria for patients with SCI-NP: (1) Patients with thoracic SCI who met the International Standards for Neurological Classification of Spinal Cord Injury revised by the American Spinal Injury Association (ASIA); (2) Clinically diagnosed with NP for more than 3 months; (3) Visual Analog Scale (VAS) score > 3; (4) Assessed by the Edinburgh Handedness Inventory questionnaire (Oldfield, 1971) as right-handed with normal muscle strength in the right hand (graded 5 on the Manual Muscle Testing scale); (5) No history of other relevant chronic pain; and (6) Able to cooperate with the examination.

Exclusion criteria: (1) Cognitive or consciousness impairment; (2) Other diseases that can cause NP, such as diabetes mellitus, herpes zoster, and tumors; (3) Other neurological or psychiatric disorders.

For patients with SCI (without NP), all recruitment criteria are consistent with those for patients with SCI-NP except for the criteria related to NP.

### 2.2 Study design

Demographic, sociological, and clinical characteristics of the subjects were recorded, including name, gender, age, injury level, and ASIA impairment scale. For patients with SCI-NP, additional pain-related assessments were conducted, including evaluations of pain location, single/multiple pain sites, pain quality, as well as assessments using the Neuropathic Pain Scale (NPS) (Yilmazer et al., 2025) and Pittsburgh Sleep Quality Index (PSQI) (Zhang et al., 2024). And information on whether they had received traditional Chinese medicine (TCM) treatment within the 3 months prior to enrollment.

**Figure 1:**
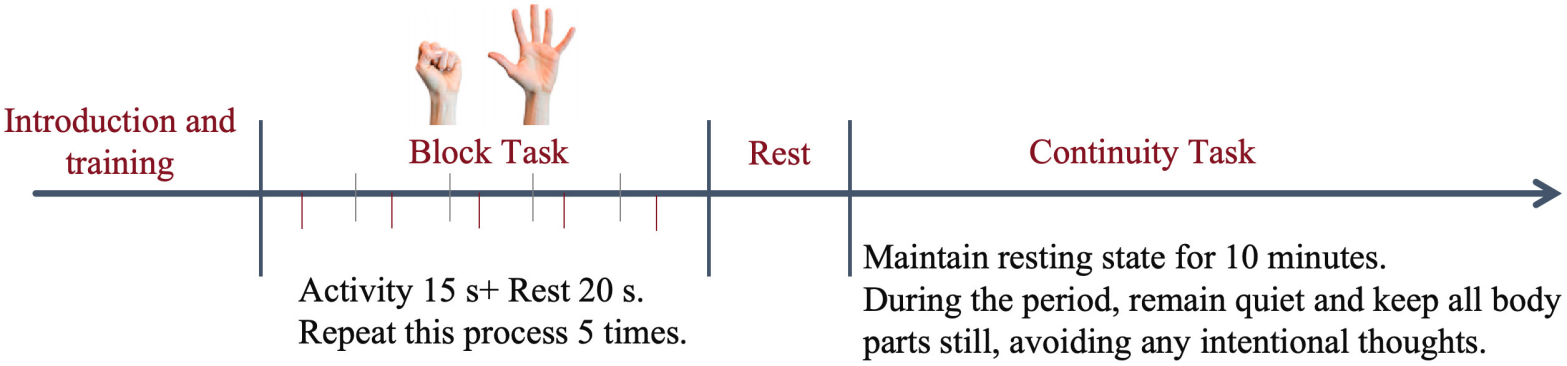
fNIRS detection protocol.

All participants were required to receive fNIRS task introduction and training before the experiment initiation. The experiment consisted of two parts: block design and continuous RS acquisition mode. The former was used to detect HbO concentration (unit: mmol/L) in each channel under block states, while the latter was used to detect brain network FC; detailed procedures and methods are shown in Figure 1.

During the RS, participants remained quiet and minimized any intentional thinking as much as possible. During the task state, they continuously performed right hand grasping and extending movements at a frequency of 30 times per minute with a target force of approximately 5 N per movement (Participants were asked to determine a force of 5N with the help of the dynamometer and practice at a frequency of 30 times per minute to ensure they could accurately complete the task as required before the formal test. No measures were taken to prevent participants from being distracted during the test, so no dynamometer was used to assist with gripping). Throughout the process, the computer provided voice prompts to the subjects at each time point according to the preset program.

The experiment was conducted in a quiet room, with only one researcher and one participant present. Participants kept their eyes closed, maintained immobility of all body parts, refrained from speaking, avoided distraction, and sat comfortably while wearing the fNIRS acquisition cap. Both hands were naturally placed on the bilateral thighs, with the right palm facing upward and the left palm facing downward (Yu et al., 2024; Üçeyler et al., 2015).

### 2.3 fNIRS device

**Figure 2:**
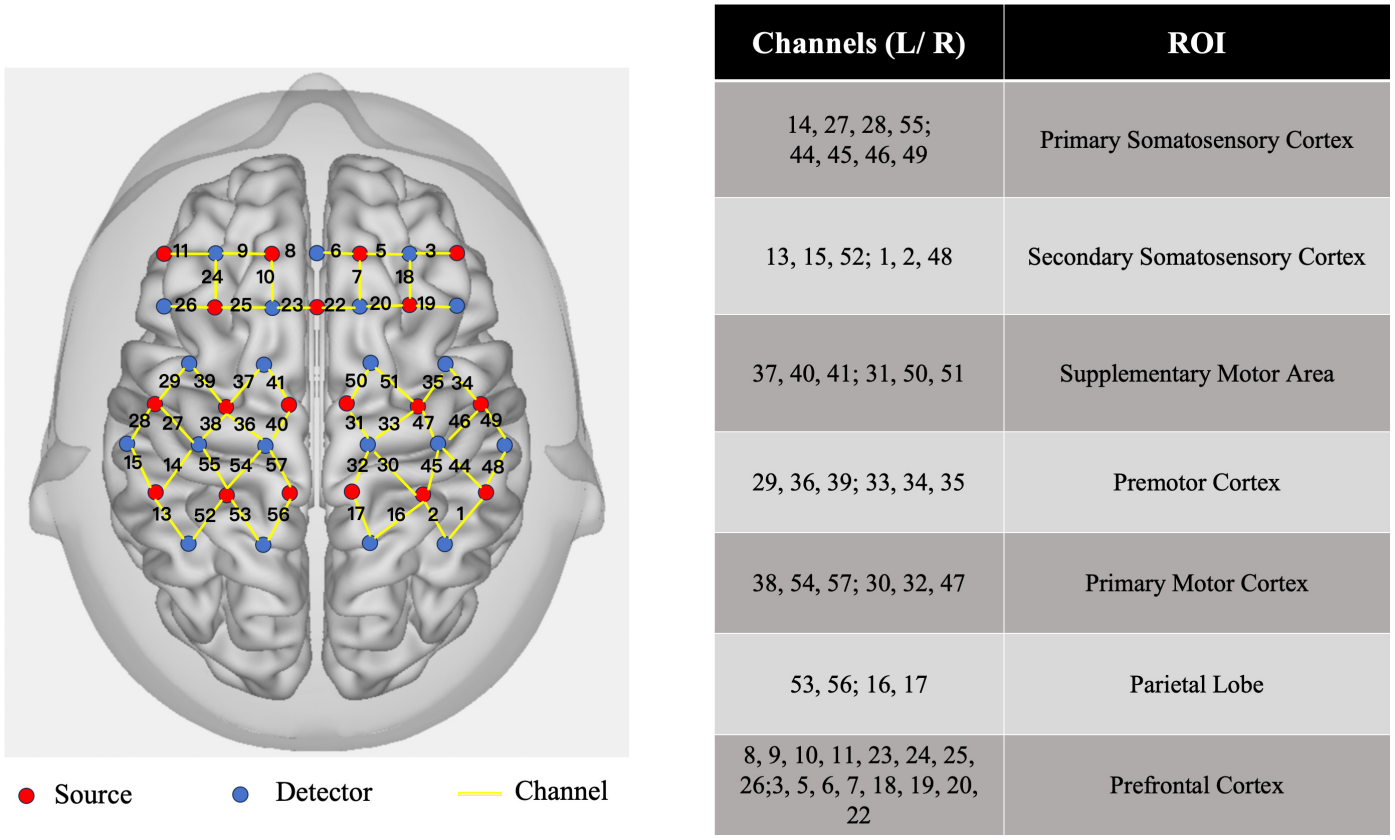
Schematic diagram of the fNIRS probe layout and ROI division (red circles represent photon emitters, blue circles represent photon detectors, and yellow solid lines represent fNIRS channels).

In this study, a multi-channel fNIRS imaging device (NirSmart, NirScan-9000A, Danyang Huichuang Medical Devices Co., Ltd., China) was used to acquire signals related to brain functional activity. The device emits near-infrared light with three different wavelengths (730, 808, and 850 nm) at a sampling frequency of 11 Hz, enabling real-time detection of changes in HbO levels in the subjects' brains. The fNIRS acquisition cap was designed based on the International 10/20 System for electrode placement. According to Brodmann's cortical functional localization, emitter probes and detector probes were placed on the scalp corresponding to specific brain regions of interest (ROIs) (Figure 2), with a channel for receiving blood oxygen signals formed between each pair of probes.

In this study, the ROIs were set as the bilateral primary sensorimotor cortex (S1), secondary sensorimotor cortex (S2), supplementary motor area (SMA), PMC, primary motor cortex (M1), parietal lobe (PL), and PFC. Specifically, there were 4 channels in the unilateral S1, 3 channels in S2, 3 channels in SMA, 3 channels in M1, 3 channels in PMC, 2 channels in PL, and 8 channels in PFC. The arrangement and number of channels in the corresponding ROIs on both sides were consistent.

### 2.4 Data preprocessing

Data preprocessing was performed using NirSpark 1.8.1 (Danyang Huichuang Medical Devices Co., Ltd., China) software (Yu et al., 2024): First, manual data quality inspection was performed to promptly exclude data segments with severe motion interference or invalid signals and concatenate the remaining valid segments; Raw light intensity signals were converted into raw optical density (OD) signals; A moving sliding window algorithm was used to identify motion artifacts in OD signals, and a spline interpolation algorithm was applied to remove these artifacts (parameters: sliding window standard deviation threshold = 6, amplitude threshold = 0.5, moving sliding window step size = 0.5, mask length = 0.5; an OD signal fluctuation exceeding ±0.1 OD was defined as an artifact; Signal quality criterion: A SNR ≥ 3 was considered acceptable, and channels with SNR < 3 were classified as invalid); A 0.01–0.1 Hz filter (Butterworth band-pass filter: 3rd-order for low-pass, 5th-order for high-pass) was used to perform band-pass filtering on OD signals, so as to eliminate noise caused by cardiac activity, respiration, Mayer waves, and systematic drift; Finally, OD signals were converted into changes in hemoglobin concentration based on the modified Beer-Lambert law (it is particularly noteworthy that high-quality data were acquired via fNIRS in this study, with no channels excluded or segments of blood oxygenation signals truncated). In this study, the HbO signal was mainly analyzed, as they have a higher signal-to-noise ratio than HbR and are more sensitive to monitoring regional cerebral blood flow (Mürner-Lavanchy et al., 2025).

### 2.5 Data processing

Data acquired in block design were analyzed using NirSpark software. The block duration was set to 35 seconds (including 15 seconds of task state and 20 seconds of resting state) [0 s, 35 s], with the baseline set to [−2 s, 0 s]. Subsequently, data from five blocks were stacked and averaged to obtain the average HbO change time series, namely the task-related hemodynamic response function, thereby calculating HbO levels in each channel during the task state and RS.

In this study, only HbO signals were used to calculate RS FC, which was also analyzed using NirSpark software. Pearson correlation coefficient r between the time series of all channels were computed to determine the FC between each pair of measured channels (Wang et al., 2020). A Fisher transformation of the r value was performed, and the Z value was used to represent its normal distribution. Further, the FC strength within each ROI, between ROIs, and across the whole brain was calculated by Z value. To control Type I errors caused by multiple comparisons, False discovery rate (FDR) correction was applied to the results of inter-regional and intra-regional FC group comparisons.

### 2.6 Statistical analysis

Data were analyzed and graphed using SPSS 26.0 (IBM Corporation, Armonk, New York, USA), MATLAB R2016a (MathWorks, Massachusetts, USA), NirSpark 1.8.1, and Prism 10.3 (GraphPad Software, San Diego, California, USA). The Shapiro-Wilk test was used to verify the normality of the data. Data conforming to a normal distribution were expressed as mean ± standard deviation. Paired *t-*test was used to assess intra-group differences. Three groups of data first underwent Levene's test to verify homogeneity of variance, followed by ANOVA. If ANOVA indicated a significant difference, *post-hoc* pairwise comparisons were performed using the least significant difference (LSD) test, with Bonferroni correction applied to control for multiple comparison errors. Meanwhile, given the small sample size of this study, Cohen's d was used for *t*-tests and partial eta-squared (η^2^) for analysis of ANOVA, to more accurately quantify the magnitude of the observed effects. *P* < 0.05 was considered statistically significant. Independent samples *t*-test was used to detect differences in brain FC between the two groups. False Discovery Rate (FDR) correction was applied to each significance level, and a significance level of *P* < 0.05 indicated a significant difference in FC. Pearson correlation coefficient was used for correlation analysis of pain indicators, and *P* < 0.05 indicated a statistically significant correlation. All statistical tests were two-tailed.

## 3 Results

### 3.1 Participants

A total of 35 participants were recruited in this study to undergo fNIRS testing, including 10 able-bodied controls (6 males and 4 females, with a mean age of 34.6 ± 8.4 years) and 25 patients with SCI. Among the patients with SCI, there were 12 patients with SCI-NP (8 males, 4 females, mean age 42.6 ± 16.5 years; detailed information is shown in Table 1), and 13 had without NP (it is referred to as “patients with SCI” in the results section; 10 males, 3 females, mean age 45.8 ± 20.0 years; detailed information is shown in Table 2). Table 3 compares the baseline characteristics of the three groups of participants.

**Table 1:** Characteristics of patients with SCI-NP.

**Patient**	**Age**	**Gender**	**Injury level**	**ASIA grade**	**BMI**	**Location of the pain**	**Multiple-site pain**	**Characteristics of pain**	**PSQI**	**NPS**	**Onset time**	**TCM treatment**
1	35	M	T10	A	25.35	Below	Yes	Prickling pain	25	37	4	Yes
2	39	M	T11	A	23.66	Both	Yes	Band-like sensation, Burning pain	28	66	10	Yes
3	58	M	T7	D	25.72	Below	No	Electric shock-like pain	15	23	7	Yes
4	55	M	T10	A	25.14	Below	Yes	Band-like sensation	13	25	5	No
5	68	M	T2	D	24.11	Below	No	Dull pain	17	20	16	No
6	29	F	T9	C	24.17	Below	No	Band-like sensation	22	31	14	Yes
7	64	M	T10	D	24.44	Both	Yes	Band-like sensation	26	35	9	Yes
8	18	M	T4	A	20.90	Below	No	Dull pain	11	20	6	No
9	39	M	T2	B	23.94	Below	Yes	Band-like sensation	16	29	4	No
10	50	F	T5	C	18.78	Below	No	Prickling pain	22	48	13	No
11	19	F	T6	A	17.31	Below	Yes	Prickling pain	19	32	15	Yes
12	37	F	T12	D	21.22	Both	Yes	Electric shock-like pain	11	25	8	No

Age was expressed in years, BMI in kg/m^2^, Onset time in months, and the results of PSQI and NPS were presented as scores. In “Injury level,” “T” stands for thoracic SCI, and the number following it indicates the specific level of the injured segment. The NPS allows patients to self-assess their own NP across ten dimensions: Intensity, Sharpness, Hotness, Dullness, Coldness, Sensitivity, Itchy, Unpleasantness, Intensity of deep pain, and Intensity of surface pain. The total score of this scale is 100 points, with 10 points for each dimension; a higher score indicates a more severe degree of NP.

**Table 2:** Characteristics of patients with SCI.

**Patient**	**Age**	**Gender**	**Injury level**	**ASIA grade**
a	61	F	T1	D
b	72	F	T11	D
c	46	M	T10	A
d	42	M	T8	D
e	20	M	T1	B
f	19	M	T11	C
g	38	M	T2	A
h	34	M	T10	A
i	65	M	T12	B
j	20	M	T10	A
k	61	M	T11	D
l	71	M	T8	D
m	75	F	T10	A

Age was expressed in years. In “Injury level,” “T” stands for thoracic SCI, and the number following it indicates the specific level of the injured segment.

**Table 3:** Baseline characteristics of the study participants.

**Characteristics**	**Health**	**SCI**	**NP**	**F/χ^2^, P**
Age	34.6 ± 8.4	45.8 ± 20.0	42.6 ± 16.5	F = 1.853, *P =* 0.173
Gender (male/female)	6/4	10/3	8/4	χ^2^ = 0.782, *P =* 0.738
ASIA grade (A/B/C/D)	-	5/2/1/5	5/1/2/4	χ^2^ = 0.739, *P =* 0.864
Injury level (T1-T6/T7-T12)	-	3/10	6/6	χ^2^ = 1.963, *P =* 0.226

### 3.2 ROI activation

**Figure 3:**
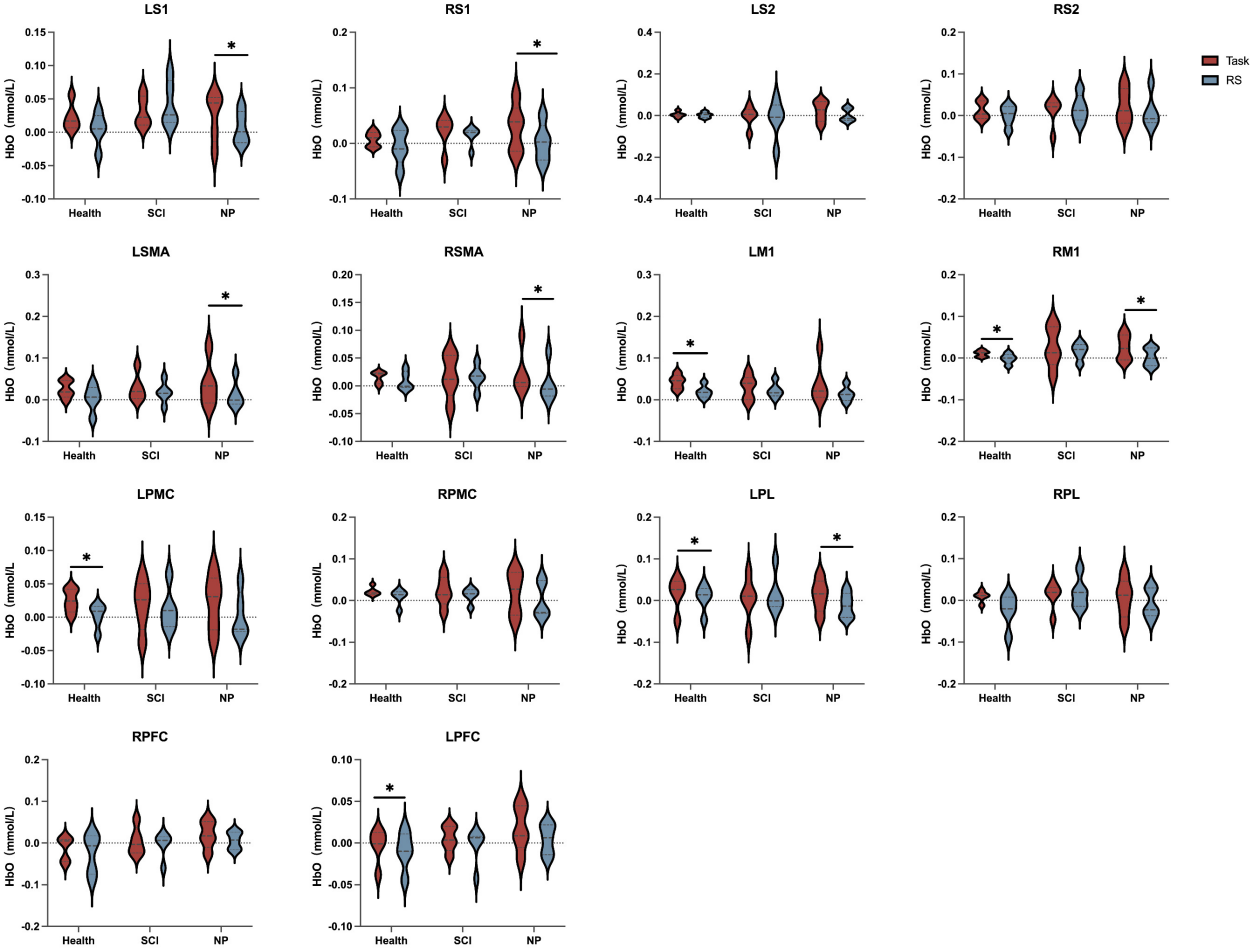
Paired-sample *t*-test was used to analyze the differences in blood oxygen changes in each ROI during Task by the three groups of subjects compared to RS, based on the activation status of ROIs; *denotes 0.01 < *P* < 0.05.

As shown in Figure 3, in the block design, compared with the RS, able-bodied controls in the task state exhibited activation in the LM1 (t = 2.408, *P* = 0.037, Cohen's d = 1.157), RM1 (t = 2.293, *P* = 0.042, Cohen's d = 0.852), LPMC (t = 2.685, *P* = 0.028, Cohen's d = 0.765), LPL (t = 2.167, *P* = 0.048, Cohen's d = 0.691), and left LPFC (t = 2.270, *P* = 0.043, Cohen's d = 0.832); patients with SCI showed no activation in any ROIs; patients with SCI-NP demonstrated the most extensive ROI activation, involving bilateral S1 (LS1: t = 2.247, *P* = 0.044, Cohen's d = 0.639; RS1: t = 2.719, *P* = 0.032, Cohen's d = 0.582), bilateral SMA (LSMA: t = 2.549, *P* = 0.041, Cohen's d = 0.714; RSMA: t = 2.564, *P* = 0.037, Cohen's d = 0.767), RM1 (t = 2.391, *P* = 0.038, Cohen's d = 1.253), and LPL (t = 2.392, *P* = 0.047, Cohen's d = 0.577). Additionally, we present the results of the paired *t*-test for HbR, which are provided for reference and supplementary purposes; see Supplementary Table S1 for details.

### 3.3 ΔHbOC of ROI

**Figure 4:**
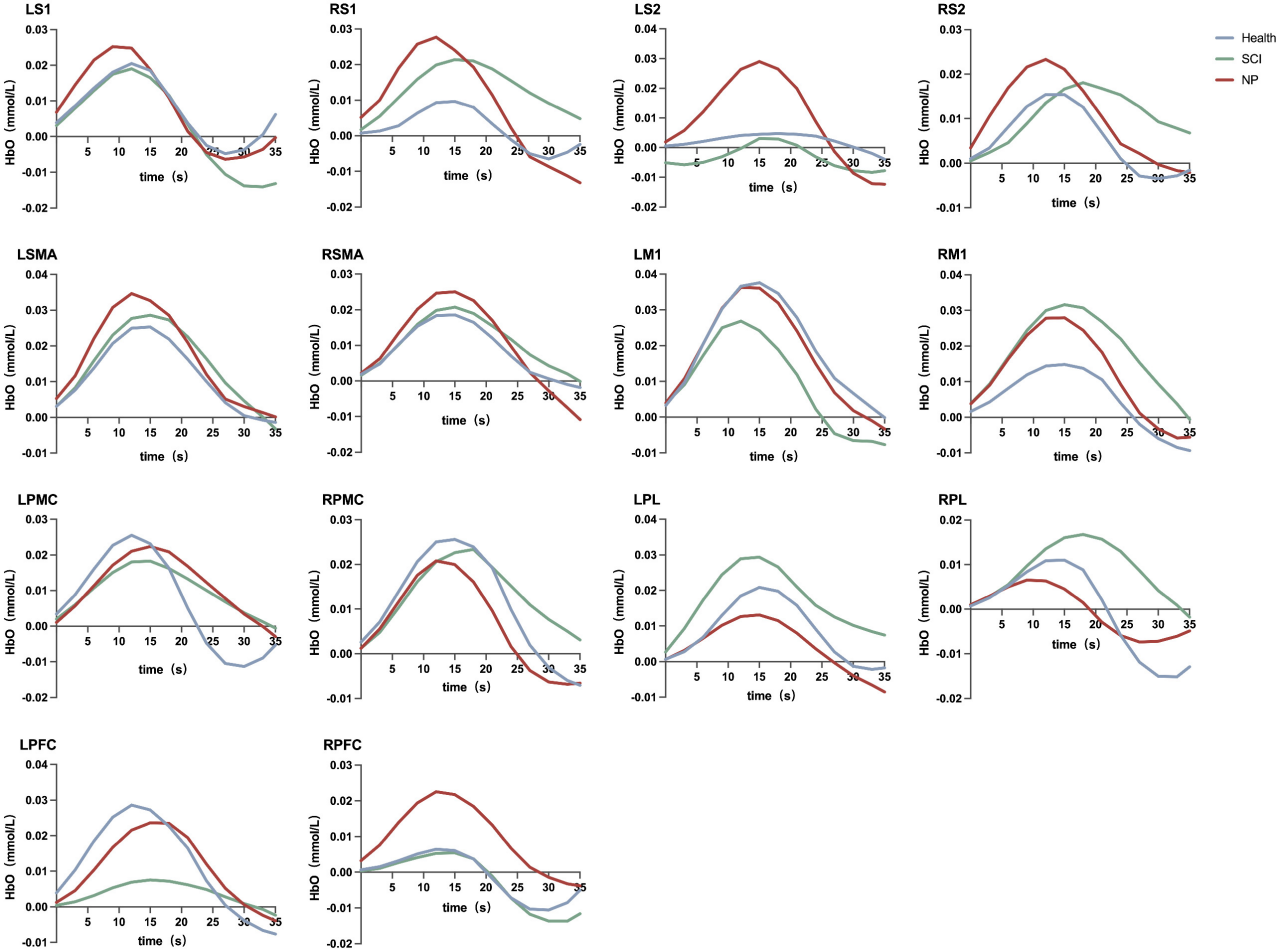
The changes in HbO concentration in each ROI during the average block design.

The degree of HbOC change in each ROI was calculated using the formula ΔHbOC = HbOC_Task_ - HbOC_RS_, as shown in Figure 4. The HbOC changes in bilateral S1, bilateral S2, bilateral SMA, and RPFC, the ΔHbOC of patients with SCI-NP exhibited a relatively higher changing trend compared with able-bodied controls and patients with SCI. In LM1, LPMC, RPMC, and LPFC, able-bodied controls showed the highest ΔHbOC.

### 3.4 FC between ROIs

**Figure 5:**
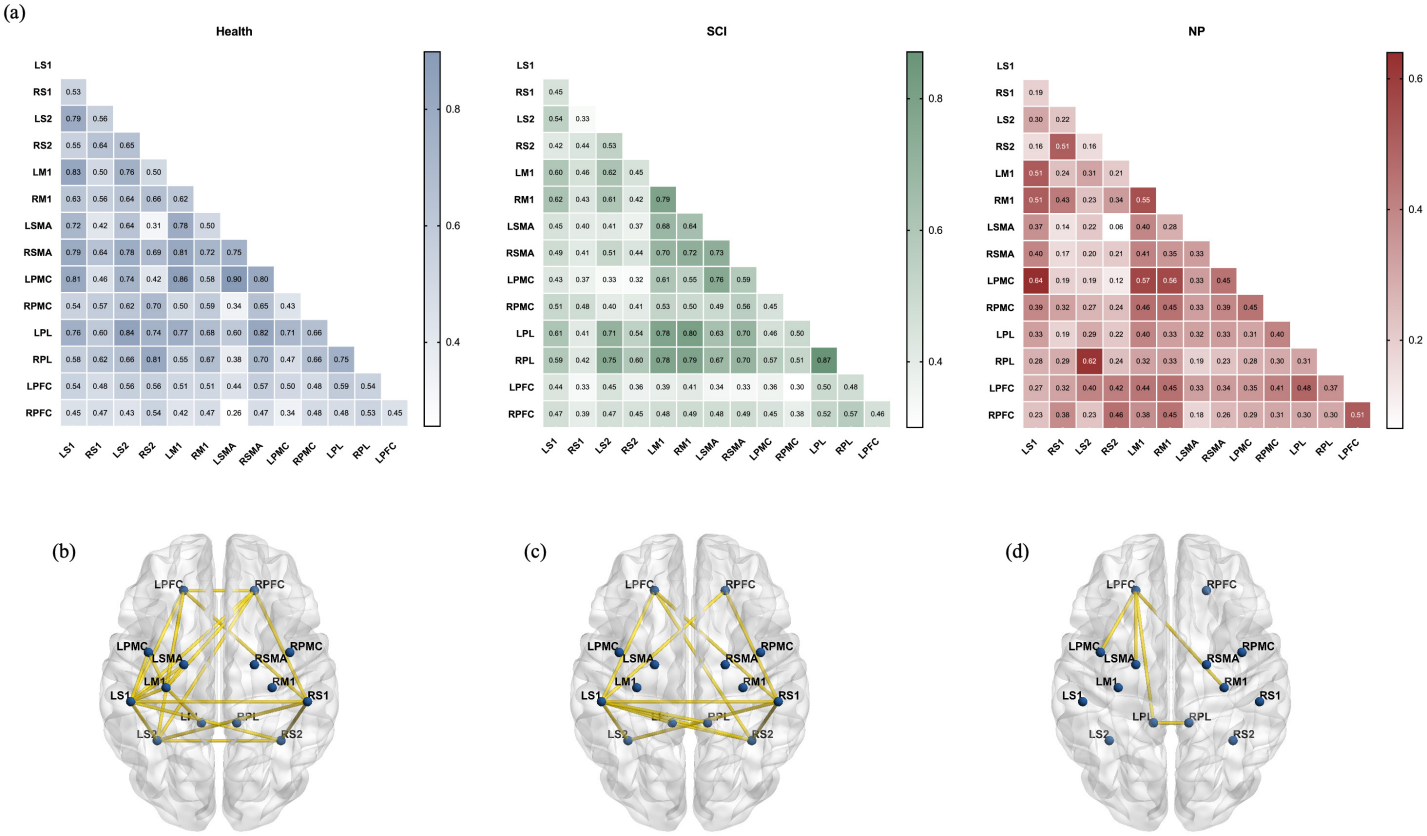
**(a)** The FC among the ROIs of the three groups of subjects was expressed using r values. **(b)** The difference of ROIs FC strength between the able-bodied controls and patients with SCI-NP under RS. The yellow line indicated that the FC strength of group health subjects was stronger than that of patients with SCI-NP (after correction by FDR, 0.01 < *P* < 0.05). **(c)** The difference of ROIs FC strength between the patients with SCI and patients with SCI-NP under RS. The yellow line indicated that the FC strength of group patients with SCI was stronger than that of patients with SCI-NP (after correction by FDR, 0.01 < *P* < 0.05). **(d)** The difference of ROIs FC strength between the able-bodied controls and patients with SCI under RS. The yellow line indicated that the FC strength of group able-bodied controls was stronger than that of patients with SCI (after correction by FDR, 0.01 < *P* < 0.05).

The FC among various ROIs was calculated as shown in Figure 5a. During the RS, the trend of FC among almost all ROIs exhibited a pattern of able-bodied controls > patients with SCI > patients with SCI-NP.

The comparison of FC among ROIs between able-bodied controls and patients with SCI-NP is presented in Figure 5b. Except for the right motor regions and bilateral PL, the FC between the remaining ROIs and the LS1 was higher in able-bodied controls than in patients with SCI-NP (0.01 < *P* < 0.05, Cohen's d = 0.582−1.233). Additionally, significant differences (0.01 < *P* < 0.05, Cohen's d = 0.748−0.956) in FC were observed between able-bodied controls and patients with SCI-NP in the following pairs: RS1 with bilateral S2 and PFC; LS2 with RS2 and bilateral PFC; between bilateral PFC; and LM1 with bilateral PFC and left motor regions. In all these cases, FC in patients with SCI-NP remained lower than that in able-bodied controls. The comparison of FC among ROIs between patients with SCI and patients with SCI-NP is displayed in Figure 5c. The FC between LS1 and bilateral S2, bilateral PFC, bilateral PL, as well as LS2 was stronger in patients with SCI than in patients with SCI-NP (0.01 < *P* < 0.05, Cohen's d = 0.568−0.817). Similarly, the same trend was observed in FC between RS1 and bilateral S2, bilateral PFC; and between RS2 and LS2, LPL, LPFC (0.01 < *P* < 0.05, Cohen's d = 0.727−1.375). Figure 5d illustrates the FC differences between able-bodied controls and patients with SCI. Significant differences (0.01 < *P* < 0.05, Cohen's d = 0.510−0.724) were only found between bilateral PL, and between LM1 and other left motor regions, RM1, as well as LPL. In all these cases, FC was higher in able-bodied controls than in patients with SCI.

### 3.5 FC within ROI

FC within each ROI is shown in Table 4. Significant differences in FC were observed across multiple ROIs among the able-bodied controls, patients with SCI, and patients with SCI-NP (*P* < 0.05). Specifically, the FC strength in the LS2, RS2, RPL, LPFC, and RPFC exhibited a trend of able-bodied controls > patients with SCI > patients with SCI-NP. Among these, the patients with SCI-NP showed significant differences compared with the able-bodied controls (additionally, LS2 and RPL also differed from the patients with SCI). In contrast, for the RM1, the trend was patients with SCI-NP > patients with SCI > able-bodied controls, with both the patients with SCI and patients with SCI-NP showing significantly higher FC than the able-bodied controls (*P* < 0.05).

**Table 4:** The correlation coefficient r value is used to represent the intra-ROI FC among the three groups of participants.

**ROI**	**Health**	**SCI**	**NP**	**F**	**P**	**η^2^**
LS1	0.824 ± 0.072	0.672 ± 0.180	0.665 ± 0.122	2.305	0.142	0.377
RS1	0.711 ± 0.131	0.529 ± 0.107	0.604 ± 0.164	2.249	0.148	0.273
LS2	0.855 ± 0.033	0.742 ± 0.091	0.518 ± 0.110^a^	20.562	< 0.001^***^	0.774
RS2	0.796 ± 0.111	0.671 ± 0.102	0.564 ± 0.116^a^	5.610	0.019^*^	0.483
LM1	0.824 ± 0.111	0.710 ± 0.167	0.639 ± 0.182	1.774	0.211	0.228
RM1	0.477 ± 0.126	0.670 ± 0.075	0.740 ± 0.035	12.256	0.001^**^	0.671
LSMA	0.824 ± 0.225	0.643 ± 0.224	0.590 ± 0.159	1.801	0.207	0.231
RSMA	0.842 ± 0.114	0.795 ± 0.093	0.719 ± 0.099	1.846	0.200	0.325
LPMC	0.852 ± 0.033	0.754 ± 0.146	0.593 ± 0.280^a^	2.550	0.119	0.298
RPMC	0.770 ± 0.117	0.538 ± 0.169^a^	0.559 ± 0.189	3.173	0.078	0.436
RPL	0.887 ± 0.056	0.783 ± 0.050	0.536 ± 0.253^ab^	7.008	0.010^*^	0.593
LPL	0.848 ± 0.107	0.765 ± 0.164	0.582 ± 0.218	3.240	0.075	0.351
LPFC	0.680 ± 0.086	0.603 ± 0.089	0.491 ± 0.130	4.212	0.041^*^	0.412
RPFC	0.673 ± 0.096	0.638 ± 0.144	0.427 ± 0.135^a^	5.473	0.020^*^	0.477

Mean ± SD; ^*^denotes 0.01 < *P* < 0.05, ^**^denotes 0.001 < *P* < 0.01, ^***^denotes 0 < *P* < 0.001; ^a^denotes after Bonferroni correction 0.01 < *P* < 0.05 compared with able-bodied controls, and ^b^denotes after Bonferroni correction 0.01 < *P* < 0.05 compared with the patients with SCI.

### 3.6 FC of the whole brain

**Figure 6:**
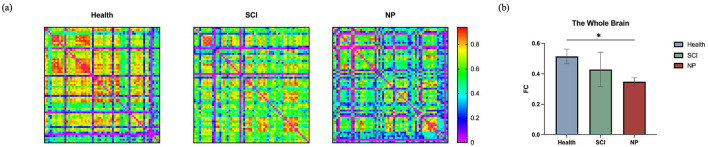
**(a)** FC of the whole brain in the three groups of subjects. **(b)** Statistical analysis results among the three groups; *denotes able-bodied controls vs. patients with SCI-NP 0.001 < *P* < 0.01.

Figure 6 illustrates the whole-brain FC characteristics among the three groups of participants. Among the three groups, the FC strength in able-bodied controls was greater than that in patients with SCI, which in turn was greater than that in patients with SCI-NP (F = 6.535, *P* = 0.012, η^2^ = 0.521), with a significant statistical difference observed between able-bodied controls and patients with SCI-NP (t = 6.238, *P* = 0.004, Cohen's d = 0.732, after Bonferroni correction *P* = 0.012).

### 3.7 Correlation of the NP-related indicators

Table 5 presents the correlations between NP-related indicators, including pain score NPS and sleep quality score PSQI, and ΔHbOC of activated ROIs, whole-brain FC, and BMI in patients with SCI-NP. Among them, the correlation coefficient between NPS and the LPL was 0.977 (*P* < 0.01), indicating a strong positive correlation; PSQI showed a strong positive correlation with the RSMA (*P* < 0.01), and significant positive correlations with the LSMA, RM1, and LPL (*P* < 0.05). No significant correlations were observed for the remaining indicators.

**Table 5:** Correlations among NP-related indicators.

**Indicator**	**Activated ROI**	**Correlation coefficient**	**P**
NPS	LS1	0.808	0.098
	RS1	0.850	0.068
	LSMA	0.588	0.297
	RSMA	0.852	0.067
	RM1	0.753	0.142
	LPL	0.977	0.004^**^
	FC- the whole brain	0.249	0.687
	BMI	−0.581	0.304
PSQI	LS1	0.737	0.155
	RS1	0.781	0.119
	LSMA	0.889	0.043^*^
	RSMA	0.975	0.005^**^
	RM1	0.934	0.020^*^
	LPL	0.936	0.019^*^
	FC- the whole brain	0.586	0.299
	BMI	−0.495	0.397

Correlation coefficient is represented by the Person r value; ^*^denotes 0.01 < *P* < 0.05, ^**^denotes 0.001 < *P* < 0.01.

## 4 Discussion

This study performed fNIRS measurements in able-bodied controls, patients with SCI and patients with SCI-NP, aiming to systematically explore the changes in characteristics of cerebral activation patterns during motor tasks and the features of RS FC in patients with SCI-NP. Meanwhile, it quantifies and evaluates the correlation between pain severity and cortical hemodynamic changes, with the purpose of providing new research perspectives and a theoretical basis for the precise assessment, objective quantification, and optimization of rehabilitation strategies for SCI-NP.

This study recruited able-bodied controls as a baseline reference, and the results showed that they exhibited activation in the LM1, RM1, LPMC, LPL, and LPFC during motor tasks. This activation pattern is generally consistent with the findings of previous studies (Yu et al., 2024). By contrast, no significant activation of any ROIs was observed in patients with SCI during the task state, suggesting that cortical excitability is suppressed in patients with SCI. This phenomenon is considered to be closely associated with the disruption of neural conduction pathways following SCI (Sun et al., 2025). The specific mechanisms can be analyzed from two aspects: on one hand, after injury to ascending sensory conduction pathways such as the spinothalamic tract and dorsal column-medial lemniscus system, peripheral somatosensory signals (e.g., limb movement feedback, external stimuli) cannot be effectively transmitted upward to the cerebral cortex. Due to the lack of sufficient afferent signal drive, the activity level of cortical neurons is downregulated, manifesting as decreased excitability (Ling et al., 2020). On the other hand, SCI can lead to the interruption of descending motor conduction pathways such as the corticospinal tract and rubrospinal tract, weakening the excitatory regulatory effect of the brain on spinal motor neurons (Chen Y. et al., 2025); meanwhile, factors such as excessive activation of local spinal interneurons may cause abnormal enhancement of inhibitory signals, which retrogradely affect the cerebral cortex via residual neural pathways (Ling et al., 2020), further exacerbating the inhibitory effect on cortical excitability.

The activation level of the cerebral cortex is closely associated with pain perception, and changes in activation patterns can directly reflect the processing and integration of pain signals (Khan et al., 2024). The results of this study showed that compared with able-bodied controls and patients with SCI (without NP), patients with SCI-NP exhibited the largest number of activated ROIs during the task state, including bilateral S1, bilateral SMA, RM1, and LPL. Their extensive activation suggests the presence of functional reorganization in the cerebral cortex of patients with SCI-NP. Previous studies have clearly indicated (Huynh et al., 2023) that structural and functional changes in the sensory cortex after SCI are one of the core mechanisms underlying the development of NP. Abnormal cortical activity induced by pain first appears in the sensory cortex responsible for primary somatosensory processing, and the significant activation of bilateral S1 regions in this study further validates this viewpoint. In addition, a meta-analysis confirmed (Solstrand Dahlberg et al., 2018) that the feedback inhibition mechanism of the cerebral cortex on the motor system is disrupted after SCI; the weakened inhibitory effect leads to enhanced activation of the motor cortex in patients with SCI-NP. The excessive activation of motor-related brain regions in patients with SCI-NP observed in this study is consistent with this conclusion.

Changes in HbO concentration can serve as a biological indicator for evaluating differences in pain processing mechanisms in pain patients (Du et al., 2023). In this study, by calculating and comparing HbO concentration changes in each ROI among the three groups of subjects, it was found that the intensity of hemodynamic changes in patients with SCI-NP was significantly higher than that in the other two groups in bilateral S1, bilateral S2, bilateral SMA, and RPFC. Pain can enhance the central sensitization effect of the central nervous system, leading to abnormally increased neuronal excitability and enhanced synaptic transmission efficiency (Volcheck et al., 2023; Donadel et al., 2021). The excessive activity in the cerebral cortex of patients with SCI-NP may indicate central sensitization. Elsehrawy et al. (2025) confirmed through a randomized controlled trial that after reducing central sensitivity in patients with knee osteoarthritis using transcutaneous vagus nerve stimulation, their pain symptoms were significantly improved. Additionally, another study (Öztürk et al., 2021) analyzed the association between changes in clinical pain variables and hemodynamic indicators in the PFC, finding that central excitability showed a corresponding decreasing trend during pain relief. Combining the conclusions of the above studies with the results of this study, it can be inferred that improving the excessive excitatory state of the cerebral cortex may serve as an effective approach to alleviate NP symptoms after SCI. This provides new insights for clinical intervention in NP and is expected to further improve patients' quality of life.

RS FC of the brain can objectively reflect the physiological functional state of the brain, as spontaneous brain activity during the RS exhibits a high degree of organizational consistency and synergy with physiological functions (Sanchez-Alonso et al., 2021). The results of this study showed that during the RS, the FC strength between almost all ROIs, within individual ROIs, and across the whole brain exhibited a trend where the FC strength in able-bodied controls was greater than that in patients with SCI, which in turn was greater than that in patients with SCI-NP. Previous studies have confirmed (Kavroulakis et al., 2021) that reduced FC levels among brain regions is an important physiological marker of impaired brain function. Based on this, it can be concluded that patients with SCI exhibit decreased FC regardless of NP comorbidity, suggesting that SCI can act on the brain through a series of mechanisms, ultimately leading to impairment of brain region function. Essentially, FC reflects the coordination and synchrony of functional activities among different brain regions. Normal brain networks possess a clear modular structure and efficient functional integration capability. However, in patients with SCI-NP, enhanced abnormal activity of the cerebral cortex can lead to weakened synchronous activity between brain regions within modules of the brain network and reduced efficiency of inter-module functional integration, ultimately manifesting as a further diffuse reduction in global FC compared to patients with SCI without NP (Jo and Perez, 2020). Even in the absence of peripheral stimulation, the pathological process of NP is still associated with long-term adaptive changes in cerebral cortex activation. Compared with both able-bodied controls and patients with SCI (without NP), patients with SCI-NP showed significant reductions in FC between multiple ROIs and within ROIs, with particularly prominent reductions in the S1, S2, and PFC.

S1 and S2 are core brain regions for somatosensory information processing. By receiving abnormal peripheral or spinal afferent signals, they induce abnormal neuronal excitability, changes in synaptic plasticity, and reorganization of functional networks, thereby participating in the perception, encoding, and modulation of pain (Goossens et al., 2019). The core function of S1 lies in the encoding of abnormal sensory signals and the regulation of their localization. After SCI, due to the interruption or disorder of peripheral afferent signals, the signals received by S1 change from ordered to disordered, resulting in patients' vague perception or mislocalization of pain location (Sanganahalli et al., 2021). Studies have confirmed that the degree of reduced FC between the DLPFC and S1 is positively correlated with the degree of hyperalgesia (Ellingsen et al., 2023). S2 is responsible for integrating pain signals transmitted by S1 with emotional and memory information, thereby constructing a complete pain experience. When the cerebral cortex of patients with SCI-NP is overexcited, abnormal signals received by S2 are excessively endowed with negative emotional significance, leading to a significant increase in patients' subjective suffering from pain—that is, even moderate-intensity pain may be accompanied by intense anxiety or fear (Krämer et al., 2008). In addition, in patients with long-term chronic pain during the resting state, the connectivity between S2 and the default network, which is responsible for self-referential thinking, is enhanced, while its connectivity with the salience network, which is responsible for detecting salient stimuli, is weakened. This makes it difficult for patients to ignore pain signals, thereby trapping them in a “pain and anxiety” vicious cycle.

The cerebral cortex plays a central role in pain perception and regulation, among which the PFC is one of the key brain regions involved in this process. This region is responsible for integrating information related to pain characteristics and individual pain experiences (Friston et al., 2013), including the sensory attributes, intensity, nature, cognitive evaluation of pain, and responses to unpleasant experiences. Meanwhile, different functional subregions within the PFC are respectively responsible for processing different aspects of pain. The DLPFC is mainly involved in pain modulation, pain catastrophizing, and placebo analgesia processes. Studies have shown (Corti et al., 2022) that in patients with chronic pain, the neural response of the DLPFC to painful stimuli is enhanced, and the FC of the default mode network formed by the DLPFC and other brain regions is impaired, manifested as a reduction in FC levels. The medial prefrontal cortex (mPFC) also participates in pain modulation and the emotional-cognitive integration of pain, and has clear anatomical connections with sensory processing brain regions (Ong et al., 2019). In addition, subregions such as the ventrolateral prefrontal cortex (VLPFC) (Zhao et al., 2021) and orbitofrontal cortex (OFC) (Shirvalkar et al., 2023) are also involved in pain regulation, prediction of chronic pain, and modulation of cold-induced pain. Although this study did not further subdivide the PFC into functional sub-regions, the results still indicate that the FC between the PFC and other ROIs, as well as within the PFC itself, is reduced in patients with SCI-NP compared with healthy participants and patients with SCI (without NP). This finding is consistent with the aforementioned conclusions regarding the involvement of the prefrontal lobe in pain regulation.

This study also used the NPS and PSQI to evaluate pain conditions in patients with SCI-NP, and integrated the evaluation results with fNIRS indicators. The findings showed that the self-assessed pain scores of patients with SCI-NP had a strong correlation with changes in HbO in the LPL, and sleep quality was associated with the motor cortex. The PL induces characteristic symptoms of pain such as chronification, distorted localization, and movement-evoked pain by impairing sensorimotor integration, spatial somatosensory perception, and coordinated regulation with the pain network (Silvestrini and Corradi-Dell'Acqua, 2023). Additionally, the impaired sleep quality in patients with SCI-NP is still considered to be associated with abnormal excitation of the cerebral cortex.

Finally, given that some patients with SCI-NP included in this study had a long history of Traditional Chinese Medicine (TCM) treatment, we further analyzed the pathogenesis and potential impacts of NP from the perspective of TCM theory. From the TCM perspective of etiology and pathogenesis, SCI is mostly caused by trauma, which tends to damage the meridians, qi, and blood, leading to qi stagnation and blood stasis; meridian obstruction prevents qi and blood from properly nourishing the zang-fu organs and limbs (Liao et al., 2023), ultimately triggering abnormal pain signal transmission and the formation of persistent NP. The core TCM theory of “pain due to malnutrition” points out that the liver governs tendons, the kidney governs bones, the brain is the “sea of marrow,” and marrow is derived from kidney essence. Long-term pain often results in prolonged illness consuming qi and blood, gradually depleting liver-kidney essence and blood; insufficient essence and blood make it difficult to nourish the tendons, bones, and cerebral marrow (Li et al., 2025; Ren et al., 2020), which may further weaken the cerebral cortex's ability to regulate pain signals and motor functions. This is potentially associated with the abnormal cortical excitation and reduced functional connectivity observed in patients with SCI-NP in this study. Currently, multiple studies have confirmed that TCM treatments have a clear effect on relieving NP secondary to other diseases (Friedemann et al., 2022; Gao et al., 2024). Based on this, future studies could further explore the improvement effect of TCM interventions on the brain function patterns of patients with SCI-NP. This would provide objective neuroimaging evidence for formulating individualized TCM treatment plans and open up new research directions for the integrated TCM-Western medicine prevention and treatment of SCI-NP.

## 5 Limitation

This study has the following limitations that need to be considered. First, the sample size recruited in this study is relatively small, and no further subgroup stratification analysis was performed based on the specific symptomatic characteristics and severity of NP, which may affect the statistical power and specificity of the study results; meanwhile, the small sample size also limits the ability to consider potential confounding factors (such as the severity of pain, age, and gender). Second, there are deficiencies in the clinical evaluation of patients with SCI-NP: there is a lack of regular and standardized follow-up assessment data of NP, which may hinder in-depth analysis of the association between pain course and brain function. Additionally, in terms of brain region localization methods, this study did not perform synchronous matching between the layout of fNIRS optical detector positions and fMRI positions, the lack of such synchronous calibration may lead to potential deviations in the localization accuracy of the ROIs defined in this study. Finally, due to technical limitations, this study did not employ established signal processing methods—including principal component analysis (PCA), independent component analysis (ICA), and common average reference (CAR)—to correct superficial physiological signals, such as hemodynamic interference from the scalp and skull. This may have compromised the accuracy and specificity of the detected cerebral hemodynamic signals. In response to the above limitations, future studies should expand the sample size, adopt stratified sampling to ensure age and gender matching between groups, conduct stratified analysis based on NPS scores to minimize confounding factor impacts, carry out standardized assessment, recording and follow-up of NP patients, and improve fNIRS signal processing techniques to further optimize study design and address existing deficiencies.

## 6 Conclusion

This study conducted a exploring analysis of the characteristics of brain functional activity among able-bodied controls, patients with SCI, and patients with SCI-NP using fNIRS. It demonstrated that SCI-NP exhibit significant abnormal excitation of the cerebral cortex and reduced FC among brain regions. Additionally, the results indicated that changes in HbO concentration can serve as a potential biomarker for the quantitative assessment of NP severity. Furthermore, leveraging its technical advantages such as portability and non-invasiveness, fNIRS is expected to become an effective tool for the objective assessment of NP, thereby providing a theoretical basis and practical support for the precision diagnosis of SCI-NP and the formulation of individualized rehabilitation strategies.

## Data Availability

The raw data supporting the conclusions of this article will be made available by the authors, without undue reservation.
