# Supplementary material for: Characteristic of cerebral hemodynamics and functional connectivity in patients with neuropathic pain after spinal cord injury: an exploratory study
Source: Front Neurosci. 2025 Dec 10;19:1699161. doi: 10.3389/fnins.2025.1699161 (PMC12728002; doi:10.3389/fnins.2025.1699161)
Supplement: Supplementary file 1 [file Table_1.docx]

Supplementary Material

# Supplementary Table

Supplementary Table 1. Presentation of paired-sample t-test results derived from analyzing cerebral activation in different subjects using HbR signals.

|  | Health |  | SCI |  | SCI-NP |  |
| --- | --- | --- | --- | --- | --- | --- |
|  | t | p | t | p | t | p |
| LS1 | 1.75 | 0.106 | -2.05 | 0.063 | -1.98 | 0.071 |
| RS1 | -1.56 | 0.144 | 0.87 | 0.401 | -1.63 | 0.129 |
| LS2 | 1.12 | 0.248 | 1.89 | 0.082 | -1.05 | 0.313 |
| RS2 | 1.33 | 0.212 | -2.12 | 0.055 | 1.65 | 0.125 |
| LSMA | -0.98 | 0.346 | -2.05 | 0.064 | 1.77 | 0.101 |
| RSMA | 1.77 | 0.069 | 1.42 | 0.182 | -1.19 | 0.255 |
| LM1 | -2.09 | 0.058 | -0.87 | 0.400 | -1.75 | 0.106 |
| RM1 | -1.83 | 0.020 | 1.04 | 0.313 | 0.69 | 0.502 |
| LPMC | -0.87 | 0.385 | -1.58 | 0.319 | 1.47 | 0.167 |
| RPMC | 1.33 | 0.204 | -2.09 | 0.052 | -1.86 | 0.087 |
| LPL | 1.75 | 0.106 | 0.58 | 0.581 | 2.11 | 0.057 |
| RPL | 1.18 | 0.302 | -1.71 | 0.114 | 0.56 | 0.585 |
| LPFC | -2.09 | 0.058 | -1.24 | 0.238 | 1.74 | 0.108 |
| RPFC | -0.79 | 0.433 | 1.45 | 0.173 | -1.36 | 0.197 |
